# Preactivation-based chemoselective glycosylations: A powerful strategy for oligosaccharide assembly

**DOI:** 10.3762/bjoc.13.207

**Published:** 2017-10-09

**Authors:** Weizhun Yang, Bo Yang, Sherif Ramadan, Xuefei Huang

**Affiliations:** 1 Department of Chemistry, Michigan State University, 578 South Shaw Lane, East Lansing, MI 48824, USA; 2 Chemistry Department, Faculty of Science, Benha University, Benha, Qaliobiya 13518, Egypt; 3 Department of Biomedical Engineering, Michigan State University, East Lansing, MI 48824, USA

**Keywords:** chemoselectivity, glycosides, preactivation, synthesis

## Abstract

Most glycosylation reactions are performed by mixing the glycosyl donor and acceptor together followed by the addition of a promoter. While many oligosaccharides have been synthesized successfully using this premixed strategy, extensive protective group manipulation and aglycon adjustment often need to be performed on oligosaccharide intermediates, which lower the overall synthetic efficiency. Preactivation-based glycosylation refers to strategies where the glycosyl donor is activated by a promoter in the absence of an acceptor. The subsequent acceptor addition then leads to the formation of the glycoside product. As donor activation and glycosylation are carried out in two distinct steps, unique chemoselectivities can be obtained. Successful glycosylation can be performed independent of anomeric reactivities of the building blocks. In addition, one-pot protocols have been developed that have enabled multiple-step glycosylations in the same reaction flask without the need for intermediate purification. Complex glycans containing both 1,2-*cis* and 1,2-*trans* linkages, branched oligosaccharides, uronic acids, sialic acids, modifications such as sulfate esters and deoxy glycosides have been successfully synthesized. The preactivation-based chemoselective glycosylation is a powerful strategy for oligosaccharide assembly complementing the more traditional premixed method.

## Review

### Introduction

Carbohydrates are widely present in nature and many of them are involved in important physiological and pathological events, such as anticoagulation, inflammation and pathogen infection [1–2]. In order to explore their biological functions, oligosaccharides with high purity are needed [3]. However, this is hampered by the limited availability of complex glycans from nature. Thus, chemical synthesis is a powerful approach to provide much needed samples to enable biological studies [4].

**Scheme 1:**
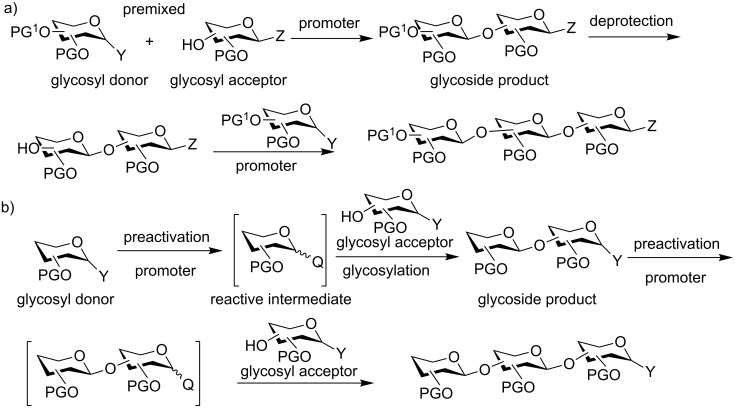
a) Traditional glycosylation typically employs the premixed approach with both the donor and the acceptor mixed together, before the promoter is added; b) the preactivation based glycosylation strategy activates the glycosyl donor in the absence of the acceptor, which temporally separates the donor activation step from acceptor glycosylation.

Traditional carbohydrate synthesis is commonly carried out from the reducing end to the non-reducing end with a glycosyl donor premixed with an acceptor. Upon the addition of a promoter to the reaction mixture, the donor is activated to glycosylate the acceptor yielding a disaccharide, which is subsequently deprotected to expose a free hydroxy group (Scheme 1a). The newly generated acceptor can be coupled with another donor and this process is repeated until the desired oligosaccharide structure is assembled. Although many oligosaccharides have been successfully produced through this approach, the traditional oligosaccharide synthesis requires multiple synthetic manipulations on oligosaccharide intermediates, which lowers the overall synthetic efficiency.

To expedite the oligosaccharide assembly process, many innovative strategies have been developed [5], such as active-latent activation [6–8], orthogonal glycosylation [9–10], reactivity-based armed-disarmed glycosylation [11–14], fluorine-supported glycosylation [15–16] and automated solid-phase synthesis [17]. All of these methods use the donor/acceptor premixed approach and preferential activation of the donor is achieved by the higher anomeric reactivity of the donor towards the promoter compared to the acceptor. In comparison, the preactivation-based iterative glycosylation is unique, where a glycosyl donor is preactivated in the absence of an acceptor to produce a reactive intermediate (Scheme 1b) [18–21]. Upon complete donor activation, the acceptor is added to the reaction mixture, which nucleophilically attacks the intermediate forming the desired glycosidic product [22–24].

With the preactivation protocol, the donor activation and acceptor glycosylation occur in two distinctive steps. As a result, a unique chemoselectivity can be achieved with preactivation. Glycosyl donors and acceptors with the same aglycon leaving group can be used enabling an iterative glycosylation, simplifying the overall synthetic design.

For a preactivation based glycosylation reaction to be successful the intermediate formed upon preactivation must be stable prior to the addition of the acceptor and yet reactive enough to quickly react with the acceptor during the glycosylation step without the need for another exogenous promoter or separation of the intermediate. Various types of glycosyl building blocks and promoter systems have been developed for preactivation. This review will be divided according to the type of glycosyl donors that can undergo a preactivation-based chemoselective glycosylation with an emphasis on thioglycosides due to their wide applicability.

### Preactivation of glycosyl sulfoxides: early success of preactivation

**Scheme 2:**
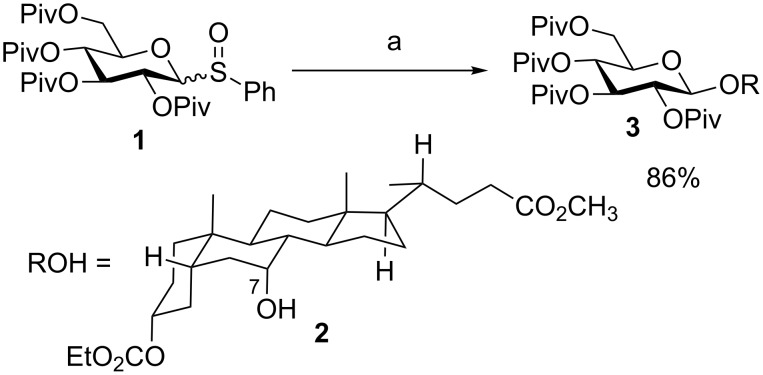
Glycosylation of an unreactive substrate. Reagents and conditions: (a) Tf_2_O, −78 °C, CH_2_Cl_2_ (DCM), then **2**, DTBMP.

One of the earliest preactivation-based glycosylation reactions utilized glycosyl sulfoxide donors for glycosylation of unreactive substrates such as steroid derivative **2** by the Kahne group [25]. The axial C-7 hydroxy group in **2** is sterically hindered due to unfavorable 1,3-diaxial interactions. The traditional premixed glycosylation gave only low yields (<30%) of the products [26]. In contrast, when glycosyl sulfoxide donor **1** was preactivated with Tf_2_O at −78 °C, followed by the addition of sterol **2** and 2,6-di-*tert*-butyl-4-methylpyridine (DTBMP) as an acid scavenger, the desired compound **3** was obtained in an excellent 86% yield (Scheme 2). While this method has not been applied to glycosyl sulfoxide as the acceptor for iterative glycosylation, this early example demonstrated the power of preactivation. Subsequently, a wide range of glycosyl donors have been explored.

### β-Glycosyl bromide-mediated iterative gycosylation of selenoglycosides

**Scheme 3:**
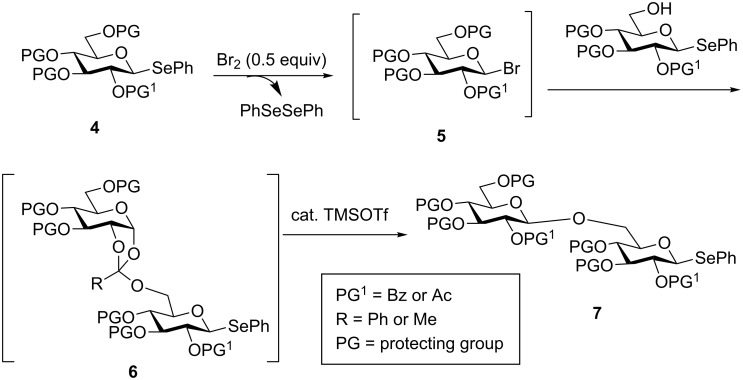
Bromoglycoside-mediated glycosylation.

**Scheme 4:**
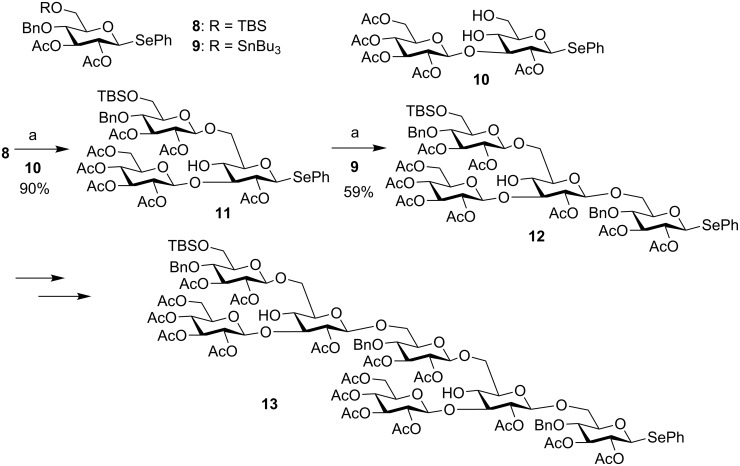
Glycosyl bromide-mediated selenoglycosyl donor-based iterative glycosylation. Reagents and conditions: (a) Br_2_, (0.5 equiv), 0 °C, CH_2_Cl_2_; then **10** or **9**, rt; then TMSOTf (0.1 equiv), 0 °C.

Yoshida and co-workers developed a preactivation-based glycosylation approach using selenoglycosides via the intermediacy of β-glycosyl bromides (Scheme 3) [27–28]. Upon the addition of 0.5 equiv of bromine, half of the selenoglycoside donor **4** would be activated to presumably form glycosyl bromide **5** accompanied by the generation of PhSeBr. PhSeBr could react with the remaining donor **4** for quantitative activation of **4**. The addition of the acceptor to the reaction mixture upon donor preactivation afforded orthoester **6**. The orthoester **6** was rearranged in situ with trimethylsilyl trifluoromethanesulfonate (TMSOTf) to disaccharide **7**, which could be subjected to bromine-promoted glycosylation for further chain elongation. As an example, preactivation of a monosaccharide **8** with bromine was followed by the addition of a bifunctional disaccharide building block **10** and subsequent TMSOTf-promoted orthoester rearrangement, producing trisaccharide selenoglycoside **11** in 90% yield (Scheme 4). Following the same reaction protocol trisaccharide **11** and glycosylated acceptor **9** lead to tetrasaccharide **12,** which was further extended to heptasaccharide **13**. This method has also been applied to generate a library of phytoalexin elicitor-active oligoglucosides [28].

A limitation of this glycosyl bromide-mediated selenoglycoside iterative glycosylation is that it is restricted to the formation of 1,2-*trans*-glycosyl linkages. Furthermore, an additional isomerization step is needed to transform the orthoester to the desired glycoside.

### Preactivation-based iterative glycosylation of 2-pyridyl glycosides

**Scheme 5:**
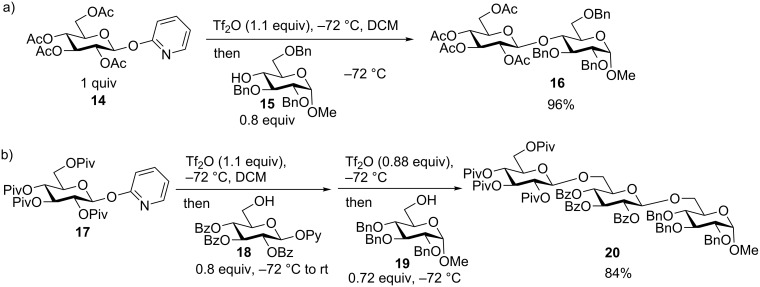
Preactivation-based glycosylation using 2-pyridyl glycosyl donors.

*O*-Unprotected 2-pyridyl glycosyl donors have been utilized in oligosaccharide synthesis [29]. The Ye group reported a preactivation protocol using protected 2-pyridyl donors [30]. The preactivation of 2-pyridyl glycoside **14** was performed using Tf_2_O as the promoter, which was followed by the addition of acceptor **15** generating disaccharide **16** in 96% yield (Scheme 5a). The acceptor could also bear a 2-pyridyl aglycon such as acceptor **18**. The preactivation-based glycosylation of donor **17** with acceptor **18** led to a disaccharide intermediate, which was then subjected to another round of Tf_2_O-mediated glycosylation leading to trisaccharide **20** in one pot (Scheme 5b). As compounds **16** and **20** have relatively simple structures, the scope of this 2-pyridyl glycosylation method will need to be established in the total synthesis of more complex oligosaccharides.

### Chemoselective dehydrative glycosylation with glycosyl hemiacetals

**Figure 1:**
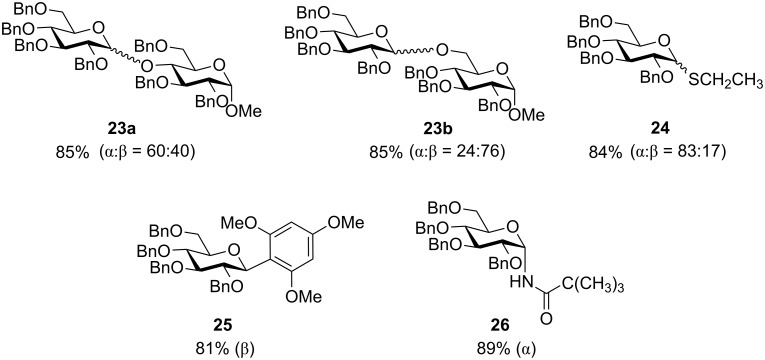
Representative structures of products formed by the preactivation-based dehydrative glycosylation of glycosyl hemiacetal.

Most glycosylation reactions require a functionalization of the anomeric position of a glycosyl donor followed by the reaction with a promoter to induce the irreversible transfer of the donor to an acceptor [31–35]. The displacement of the anomeric hydroxy group of a glycosyl hemiacetal by an acceptor for dehydrative glycosylation is an interesting alternative as glycosyl hemiacetals are often undesired side products in glycosylation reactions due to the competitive reaction with trace amounts of water present in the reaction mixture. The Gin group established a preactivation glycosylation procedure using glycosyl hemiacetals [36]. As an example, the hemiacetal donor **21** was preactivated with Tf_2_O and diphenyl sulfoxide (Ph_2_SO) at −40 °C. This was followed by the addition of the acceptor isopropyl alcohol, affording glycoside **22** in 86% yield (α:β = 27:73, Scheme 6). This glycosylation strategy can be applied to a variety of glycosyl acceptors, including oxygen, sulfur, carbon and nitrogen nucleophiles (Figure 1) [36]. Even the unreactive *N*-(trimethylsilyl)trimethylacetamide could be efficiently glycosylated to afford the corresponding glycosyl amide **26**.

**Scheme 6:**
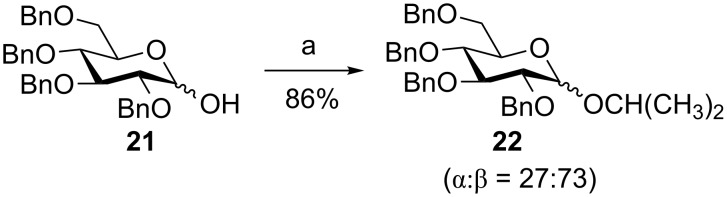
Chemoselective dehydrative glycosylation. Reagents and conditions: (a) Ph_2_SO, Tf_2_O, 2-chloropyridine, then (CH_3_)_2_CHOH, −40 °C.

**Scheme 7:**
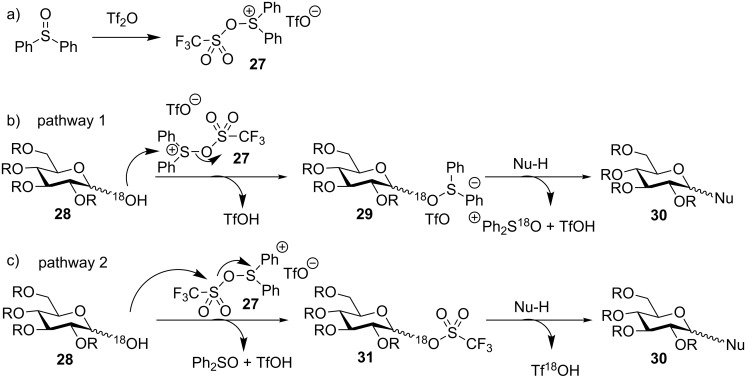
Possible mechanism for the dehydrative glycosylation. (a) Formation of diphenyl sulfide bis(triflate) (**27**) as the promoter for glycosyl hemiacetal activation; (b) pathway 1 and (c) pathway 2 as potential mechanisms for glycosyl hemiacetal activation.

Two possible reaction pathways have been proposed for this dehydrative glycosylation (Scheme 7) [37]. Upon mixing diphenyl sulfoxide and triflic anhydride, diphenyl sulfide bis(triflate) (**27**) is formed in situ (Scheme 7a). In pathway 1, hemiacetal **28** could attack the sulfonium center of diphenyl sulfide bis(triflate) (**27**) to give the glycosyl oxosulfonium intermediate **29**, which subsequently glycosylated the acceptor to yield the product **30** (Scheme 7b). Alternatively, in pathway 2, hemiacetal **28** could attack the sulfonyl center of diphenyl sulfide bis(triflate) (**27**) to give the glycosyl triflate intermediate **31**, followed by glycosylation to give **30** (Scheme 7c). To distinguish between these two possibilities, an ^18^O-labeling study was carried out by subjecting ^18^O-labeled hemiacetal **28** to the glycosylation conditions. Indeed, ^18^O-labeled diphenyl sulfoxide was detected in the reaction mixture as the main ^18^O-labeled compound, which suggested pathway 1 was the major reaction mechanism.

**Scheme 8:**
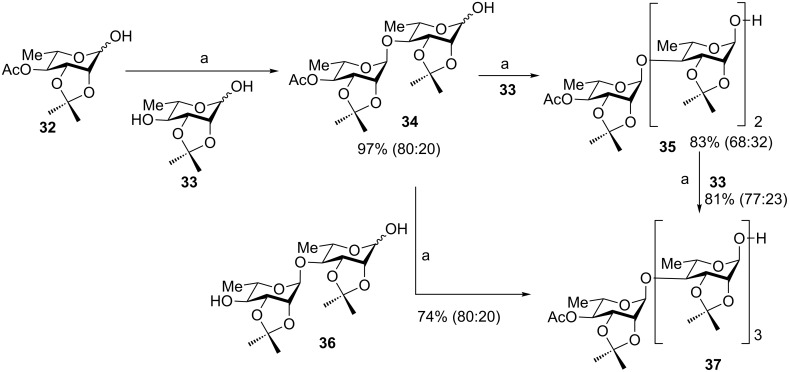
Chemoselective iterative dehydrative glycosylation. Reagents and conditions: (a) Ph_2_SO, Tf_2_O, 2,4,6-tri-*tert*-butylpyrimidine (TTBP), −78 °C to −40 °C; then acceptor.

The hemiacetal donor can be utilized in iterative glycosylation (Scheme 8) [21]. Donor **32** was preactivated by Ph_2_SO and Tf_2_O, followed by the addition of glycosyl hemiacetal **33** with one of its hydroxy groups free available as the acceptor producing disaccharide **34**. The regioselectivity is presumably due to the higher nucleophilicity of the alkyl hydroxy group than that of the hemiacetal hydroxy group. This process can be repeated for chain elongation without the need for any protective group manipulation or aglycon adjustment. Using this method, the 1,4-α-linked tetrasaccharide **37** was prepared in good overall yield.

**Scheme 9:**
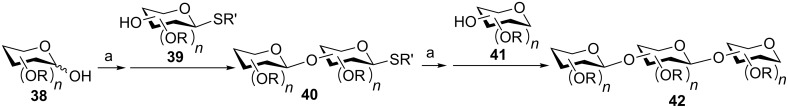
Chemoselective iterative dehydrative glycosylation. Reagents and conditions: (a) Ph_2_SO, Tf_2_O, −40 °C.

**Scheme 10:**
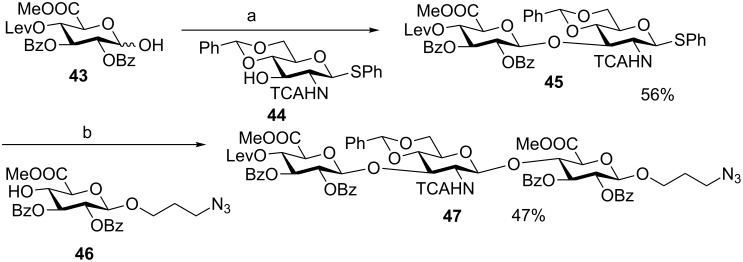
Chemical synthesis of a hyaluronic acid (HA) trimer **47**. Reagents and conditions: (a) Ph_2_SO, TTBP, CH_2_Cl_2_, −60 °C, then Tf_2_O, **44**, −60 °C to 0 °C; (b) Ph_2_SO, TTBP, CH_2_Cl_2_, −60 °C, then Tf_2_O, **46**, −60 °C to 0 °C.

Inspired by Gin’s work, van der Marvel and co-workers developed a sequential glycosylation strategy by combining hemiacetal and thioglycosyl building blocks as illustrated in Scheme 9 [38]. The hemiacetal donor **38** was preactivated with Ph_2_SO and Tf_2_O, and reacted with a bifunctional thioglycosyl acceptor **39** to form disaccharide **40**. Interestingly, thioglycoside **40** could also be activated by Ph_2_SO/Tf_2_O. The subsequent addition of acceptor **41** to the reaction mixture furnished trisaccharide **42**. This approach was applied to the synthesis of hyaluronic acid (HA) oligomers [39]. The sequential reaction of building blocks **43**, **44** and **46** led to HA trisaccharide **47** (Scheme 10). The modest overall yield of 26% for the two glycosylation reactions was attributed to the formation of orthoester and oxazolidine side products due to the basic reaction conditions, which were needed to neutralize the acid formed during glycosylation and to avoid the cleavage of the acid-labile benzylidene protective group.

**Figure 2:**
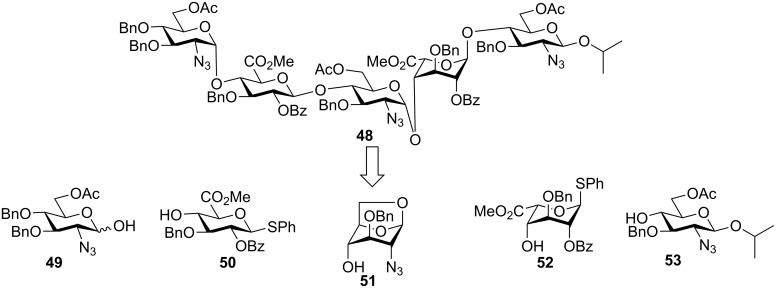
Retrosynthetic analysis of pentasaccharide **48**.

**Scheme 11:**
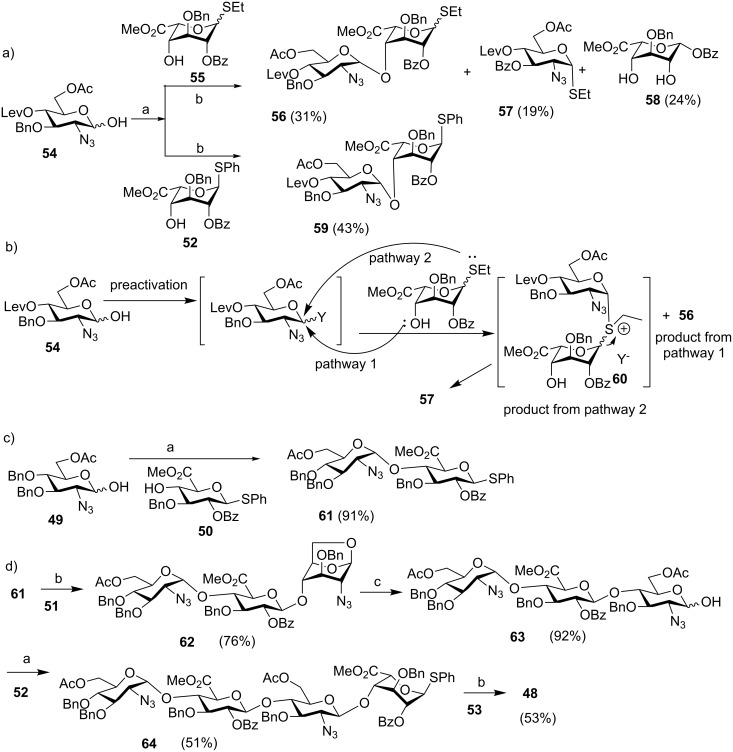
Effects of anomeric leaving groups on glycosylation outcomes. Reagents and conditions: (a) Ph_2_SO, Tf_2_O, TTBP, CH_2_Cl_2_, −40 °C; then acceptor, −40 °C to rt, (b) 1-(benzenesulfinyl)piperidine, Tf_2_O, CH_2_Cl_2_, −60 °C, then acceptor, (c) 10% trifluoroacetic acid in Ac_2_O, 0 °C to rt, then 6% piperidine in THF.

The van der Marel group further applied their strategy to the synthesis of heparin (HP) and heparan sulfate (HS), which are more complex members of the glycosaminoglycan family [40]. A pentasaccharide **48** was chosen as the synthetic target (Figure 2). A major challenge of HP and HS synthesis lies in the coupling of an azido glucoside with a uronic acid in an α-selective fashion. A variety of azido hemiacetal glucoside donor and uronic acid thioglycosyl acceptor pairs were screened under preactivation conditions. The anomeric leaving groups of the acceptors had significant impacts on the glycosylation outcomes (Scheme 11a). When donor **54** was utilized to glycosylate iduronic acid **55**, disaccharide **56** was obtained only in 31% yield along with aglycon transfer products, **57** (19%) and **58** (24%). The modest yield of the desired glycoside product resulted from the lower nucleophilicity of 4-OH as compared to the thioethyl moiety, which could compete with the nucleophilic attack by the 4-OH leading to aglycon transfer (Scheme 11b). In contrast, when thiophenyl glycoside **52** was used as the acceptor, no aglycon transfer product was isolated and disaccharide **59** was obtained in 43% yield. The improvement presumably resulted from the lower nucleophilicity of the thiophenyl moiety due to the steric as well as electronegative effects of the phenyl group. The hemiacetal donor **49** glycosylated the thiophenyl glucuronate acceptor **50** in an excellent 91% yield using the preactivation protocol (Scheme 11c). The successful preparation of disaccharides **61** and **59** paved the way for the synthesis of protected heparin pentasaccharide **48** (Scheme 11d).

### Preactivation-based chemoselective glycosylation of thioglycosides

**Scheme 12:**
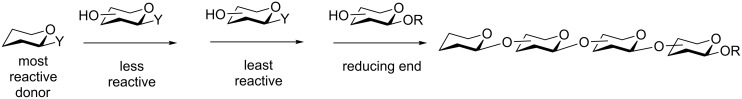
Reactivity-based one-pot chemoselective glycosylation.

Thioglycosides are one of the most commonly utilized building blocks due to their high stabilities under a wide range of synthetic transformations commonly encountered in building block preparation [41]. At the same time, mild promoters are available for thioglycoside activation. The anomeric reactivities of thioglycosides towards glycosylation can be significantly influenced by the protective groups on the glycan ring as well as the size and nucleophilicity of the thioether aglycon [42–44]. Extensive studies on how to fine tune anomeric reactivities culminated in the establishment of the powerful reactivity-based chemoselective glycosylation method [11]. In this strategy, a thioglycosyl donor with high anomeric reactivity is mixed together with a bifunctional thioglycosyl acceptor with lower anomeric reactivity (Scheme 12). Upon the addition of a promoter, the donor is preferentially activated to glycosylate the acceptor. The resulting disaccharide can then be utilized directly as a donor to react with another bifunctional thioglycoside with even lower anomeric reactivity. When building blocks with suitable anomeric reactivities are selected, multiple glycosylation reactions can be carried out in one pot without the need for synthetic manipulations or purification of the advanced oligosaccharide intermediates. This strategy, which has been covered in other reviews [23,42], has been applied to successful synthesis of a range of complex oligosaccharides including human milk oligosaccharides [45], an embryonic stem cell surface carbohydrate marker Lc4 [46], Globo-H hexasaccharide [47], and heparin-like oligosaccharides [48].

A significant drawback of the reactivity-based chemoselective glycosylation method is the requirement that the glycosyl donor must bear higher anomeric reactivities than the acceptor for preferential donor activation. As a result, extensive protecting group manipulations have to be carried out to prepare building blocks with the required anomeric reactivities. Furthermore, the relative anomeric reactivity values of a building block can vary depending on the structures of acceptors and reaction condition [44], presenting challenges in accurately predicting the reaction outcome.

**Scheme 13:**
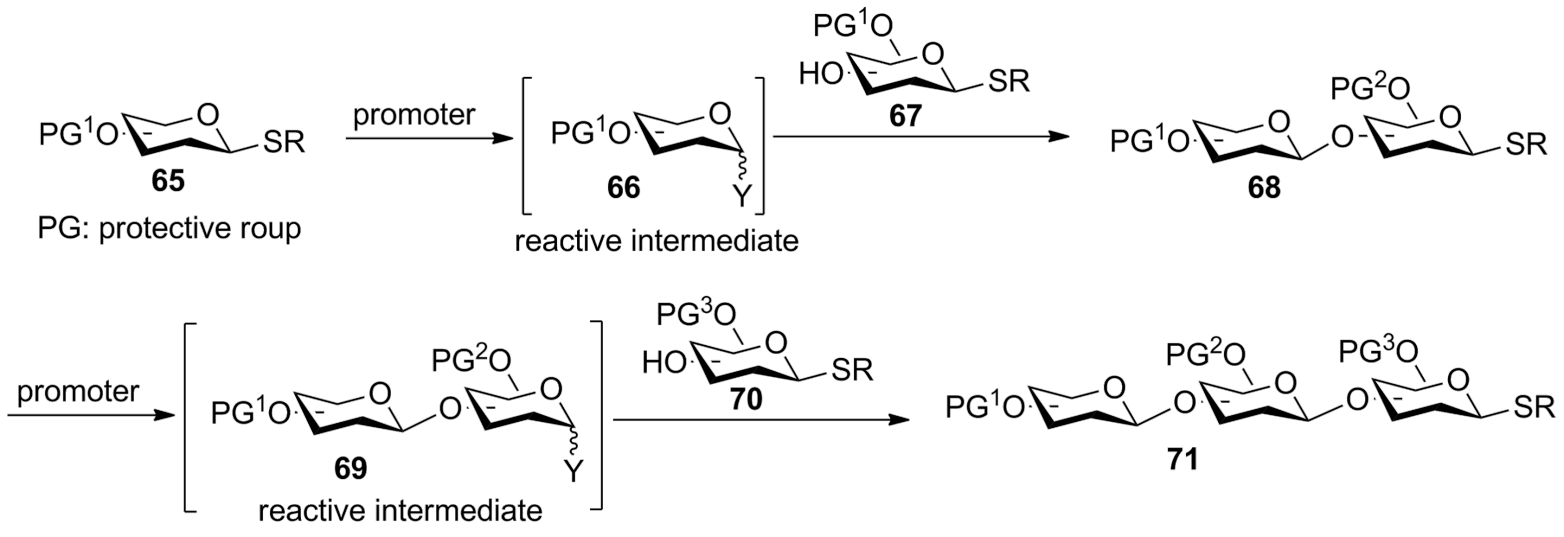
Preactivation-based iterative glycosylation of thioglycosides.

The aforementioned drawbacks of the reactivity-based chemoselective glycosylation can be overcome through preactivation. Under the preactivation protocol, a thioglycosyl donor is activated in the absence of an acceptor to form a reactive intermediate (Scheme 13). Upon complete donor activation, a thioglycosyl acceptor is added, which reacts with the intermediate to form the desired glycoside without the need for additional promoter. The resulting disaccharide bears a thioether aglycon, which can undergo another round of preactivation and glycosylation for rapid chain extension. As donor activation and acceptor glycosylation are carried out in two distinct steps, the preactivation strategy obviates the requirement that the glycosyl donor must have a higher anomeric reactivity than the acceptor for preferential activation, bestowing greater flexibilities in building block design.

A key consideration in designing successful preactivation-based thioglycoside glycosylation is the promoter. It needs to be able to activate a wide range of donors stoichiometrically rather than catalytically to avoid an undesired activation of the acceptor or the product. Many thiophilic activators have been tested, which include *p*-TolSCl/AgOTf [18], *N*-iodosuccinimide (NIS)/TMSOTf [18], dimethyl(methylthio)sulfonium triflate (DMTST) [18], 1-(benzenesulfinyl)piperidine (BSP)/Tf_2_O [18–19,49], *S*-(4-methoxyphenyl)benzene-thiosulfinate (MBPT)/Tf_2_O [50], Ph_2_SO/Tf_2_O [36,51], *O*,*O*-dimethylthiophosphonosulfenyl bromide (DMTPSB)/AgOTf [52], and 4-(benzenesulfinyl)morpholine (BSM)/Tf_2_O [53].

**Scheme 14:**
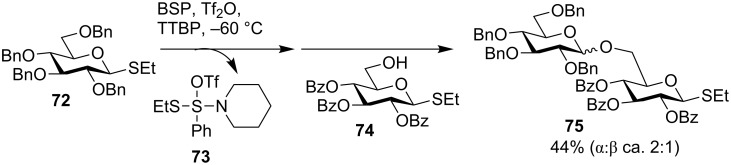
BSP/Tf_2_O promoted synthesis of **75**.

The combination of BSP/Tf_2_O [19,49] has been utilized as the promoter for iterative oligosaccharide synthesis including oligoglucosamine library [20], oligomannan [54] and Lewis^a^ trisaccharide [55]. During their synthesis, van der Marel and co-workers [19] found that with BSP/Tf_2_O promoter, the glycosylation of donor **72** and acceptor **74** gave a moderate yield of 44% (α:β = 2:1) of the desired product **75** (Scheme 14). This was attributed to the formation of (*N*-piperidino)phenyl(*S*-thioethyl)sulfide triflate (**73**) from the reaction of BSP/Tf_2_O with the thioglycosyl donor. The sulfide triflate **73** could activate the thioglycoside product, which provides a possible explanation for the modest yield. To avoid the side reaction caused by **73**, triethyl phosphite was added as a scavenger to quench **73**, which enhanced the glycosylation yield to 78%.

**Scheme 15:**
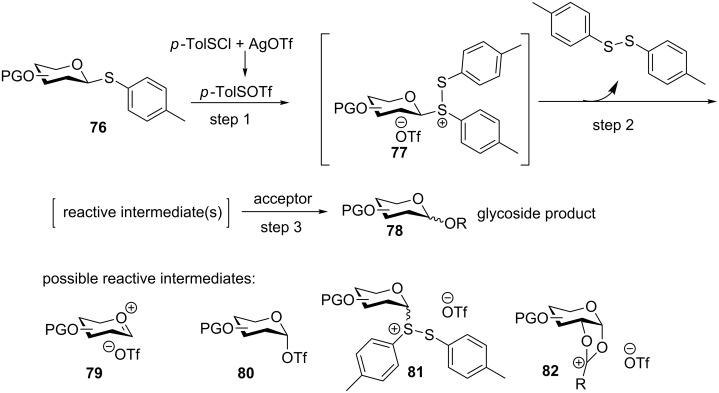
Proposed mechanism for preactivation-based glycosylation strategy.

The need for triethyl phosphite to prevent the undesired acceptor/product activation precludes the possibility of carrying out multiple glycosylation reactions in one pot using BSP/Tf_2_O. Other promoter systems such as NIS/TMSOTf, Ph_2_SO/Tf_2_O and BSM/Tf_2_O have similar complications due to the formation of thiophilic or nucleophilic side products following donor activation. Through extensive experimentation, Huang, Ye and co-workers successfully developed an iterative one-pot glycosylation strategy using the *p*-TolSCl/AgOTf promoter system and *p*-tolyl thioglycosides as building blocks [18]. A possible mechanism for this glycosylation has been proposed (Scheme 15). Addition of *p*-TolSCl to the mixture of donor **76** and AgOTf forms *p*-TolSOTf, a powerful electrophile that can electrophilically add to the anomeric sulfur atom of **76** forming disulfonium ion **77** (step 1 in Scheme 15). After ejection of the ditolyl disulfide, **77** can evolve into several reactive species, such as oxocarbenium ion **79**, α-triflate **80**, disulfonium ion **81**, and dioxalenium ion **82**. The nucleophilic attack of the intermediate by a thioglycosyl acceptor would generate the desired glycoside **78**.

**Figure 3:**
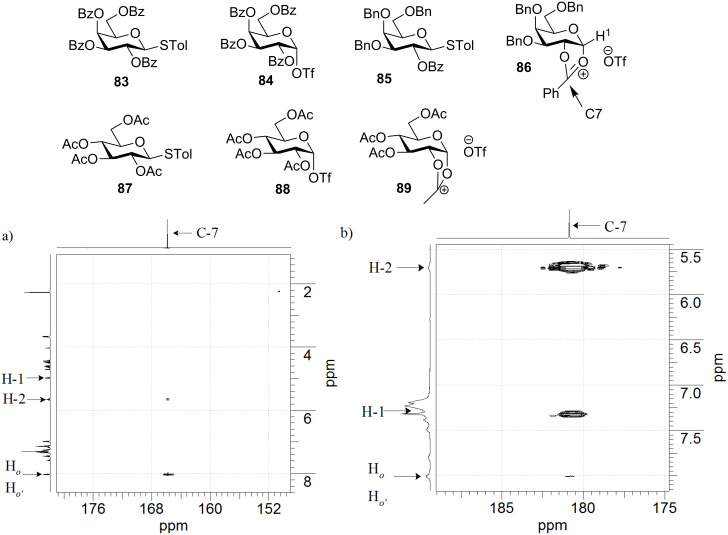
The preactivations of glycosyl donors **83**, **85** and **87** were investigated by low temperature NMR, which gave **84**, **86**, **88**/**89** as dominant intermediates, respectively. gHMBC (CDCl_3_, 600 MHz) of donor **85** a) before and b) after preactivation at −60 °C. The correlation peak emerged after activation between C-7 and H-1 supports the structure of the dioxalenium ion **86** formed from preactivation.

**Scheme 16:**
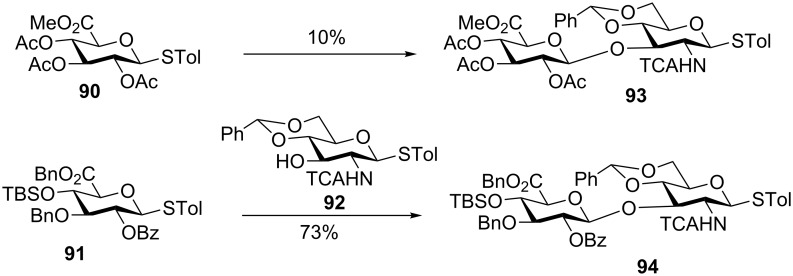
The more electron-rich glycosyl donor **91** gave a higher glycosylation yield than the glycosyl donor **90** bearing more electron-withdrawing acyl protective groups.

Pioneered by Crich and co-workers, low temperature NMR studies have been found to be a powerful approach to analyze intermediates formed during glycosylation reactions [56]. To determine the dominant intermediate in preactivation of thioglycosides, low-temperature an NMR experiments were carried out following donor activation [57]. It was determined that with perbenzoylated donor **83**, the α-glycosyl triflate **84** was formed as the major intermediate [56,58–59]. When the more electron-rich donor **85** was preactivated, the dioxalenium ion **86** via the participation of the 2-benzoyl (Bz) group was found as the dominating species from NMR analysis (Figure 3) [57]. Interestingly, when **87** was preactivated, two major intermediates were produced (α-triflate **88** and dioxalenium ion **89**). The different outcome upon preactivation can be explained in terms of different electron-withdrawing properties of the protective groups present in these three donors. For **83**, the Bz group greatly disfavors the formation of a positively charged dioxalenium ion while the electron-donating benzyl (Bn) group can stabilize the dioxalenium ion. Donor **87** presents an intermediate case. The absence of the disulfonium ion **81** following the donor activation confirms that the disulfide does not significantly impact the structure of the intermediates. The more electron-rich glycosyl donors were found to give higher yields in glycosylation, especially with unreactive and electron-poor secondary acceptors. A representative example is shown in Scheme 16. This was rationalized by higher reactivities of the dioxalenium ion than glycosyl triflate towards nucleophilic attack by the acceptor.

**Scheme 17:**
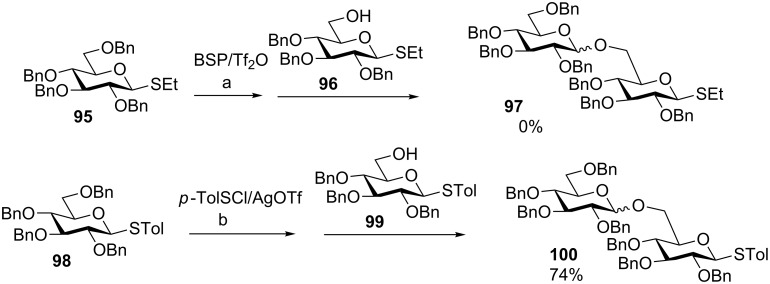
Comparison of the BSP/Tf_2_O and *p*-TolSCl/AgOTf promoter systems in facilitating the preactivation-based thioglycoside glycosylation. Reagents and conditions: (a) BSP, Tf_2_O, CH_2_Cl_2_, TTBP, −60 °C; then **96**, and triethyl phosphite; (b) *p*-TolSCl/AgOTf, −60 °C; then **99**.

*p*-TolSCl/AgOTf is a superior promoter system for the preactivation-based thioglycoside glycosylation. Some reactions that failed with the BSP/Tf_2_O promoter could be successfully performed with similar substrates using *p*-TolSCl/AgOTf (Scheme 17). This is presumably due to the inertness of the ditolyl disulfide side product from *p*-TolSCl/AgOTf promoted activation, which does not interfere with glycosylation.

**Scheme 18:**
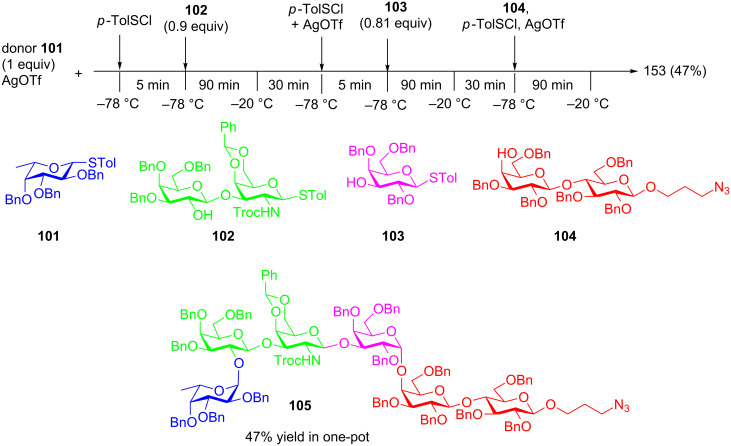
One-pot synthesis of Globo-H hexasaccharide **105** using building blocks **101**, **102**, **103** and **104**.

The *p*-TolSCl/AgOTf-promoted preactivation glycosylation has been successfully applied to the total synthesis of complex oligosaccharides including those containing both 1,2-*cis* and 1,2-*trans* linkages, branching sequences and sulfate esters. For example, a four component preactivation-based one-pot synthesis was designed to synthesize Globo-H, an important tumor-associated carbohydrate antigen (Scheme 18) [60]. Globo-H hexasaccharide **105** was prepared within 7 hours in an excellent overall yield of 47% from the sequential one-pot reaction of **101**, **102**, **103** and **104**. Compared to the automated solid-phase synthesis of Globo-H [61], the solution-based preactivation-based synthesis gave a higher overall yield for glyco-assembly (47% vs 30%) without the need for large excess (5–10 equiv) of building blocks.

**Scheme 19:**
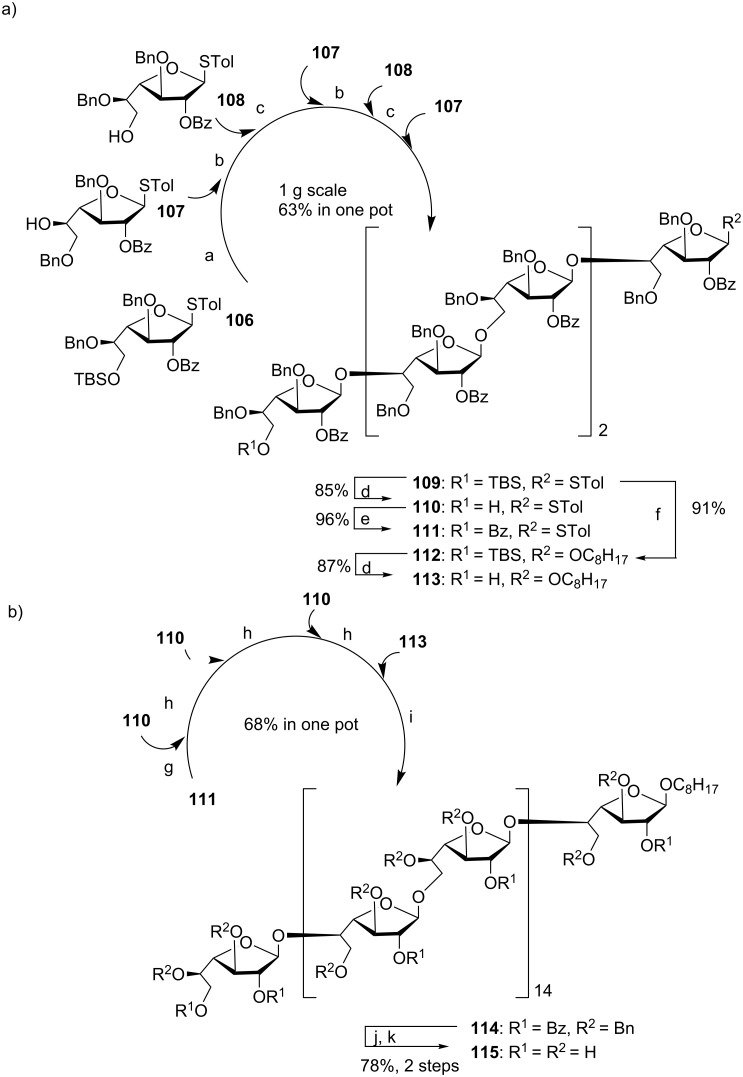
Synthesis of (a) oligosaccharides **109–113** towards (b) 30-mer galactan **115.** Reagents and conditions: (a) TTBP, 4 Å MS, CH_2_Cl_2_, *p*-TolSCl, AgOTf, then **107**, −78 °C to rt; (b) *p*-TolSCl, AgOTf, then **108**, −78 °C to rt; (c) *p*-TolSCl, AgOTf, then **107**, −78 °C to rt; (d) HF-pyridine, THF/H_2_O (10:1), 35 °C; (e) Bz_2_O, DMAP, pyridine, CH_2_Cl_2_, reflux; (f) *p*-TolSCl, AgOTf, TTBP, 1-octanol, 4 Å MS, CH_2_Cl_2_, −78 °C; (g) TTBP, 4 Å MS, CH_2_Cl_2_, *p*-TolSCl, AgOTf, then **110**, −78 °C to rt; (h) *p*-TolSCl, AgOTf, then **110**, −78 °C to rt; (i) *p*-TolSCl, AgOTf, then **113**, −78 °C to rt; (j) NaOCH_3_, CH_3_OH/CH_2_Cl_2_ (2:1); (k) Pd/C, H_2_, EtOAc/THF/1-PrOH/H_2_O (2:1:1:1).

**Figure 4:**
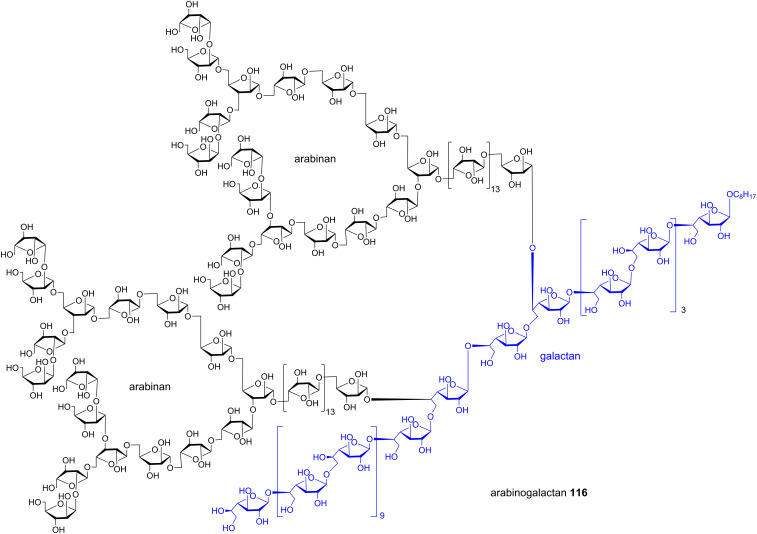
Structure of mycobacterial arabinogalactan **116**.

Recently, using a series of highly efficient preactivation-based glycosylation reactions, Ye and co-workers synthesized a mycobacterial arabinogalactan [62], which is composed of 30 D-galactofuranose residues (Gal*f*_30_) linked with two arabinan chains each containing 31 D-arabinofuranose residues (Ara*f*_31_). Both Gal*f*_30_ and Ara*f*_31_ fragments were prepared starting from monosaccharide building blocks. As an example, a six component preactivation-based glycosylation using the *p*-TolSCl/AgOTf promoter system and three monosaccharide building blocks (**106–108**) led to the formation of hexasaccharide **109** in an excellent 63% yield in one pot on a gram scale (Scheme 19a). This is the largest number of glycosylation reactions that have been performed in one pot to date. Further iterative five-component one-pot glycosylation (**111 + 110 + 110 + 110 + 113**) successfully produced protected Gal*f*_30_ 30-mer **114** in 68% yield (Scheme 19b). Following similar reaction protocols, Ara*f*_31_ was prepared, which upon glycosylation of a Gal*f*_30_ diol acceptor and deprotection, led to arabinogalactan 92-mer **116** (Figure 4) [62]. This is the largest synthetic glycan that has ever been produced, highlighting the power of the preactivation-based glycosylation strategy.

**Figure 5:**
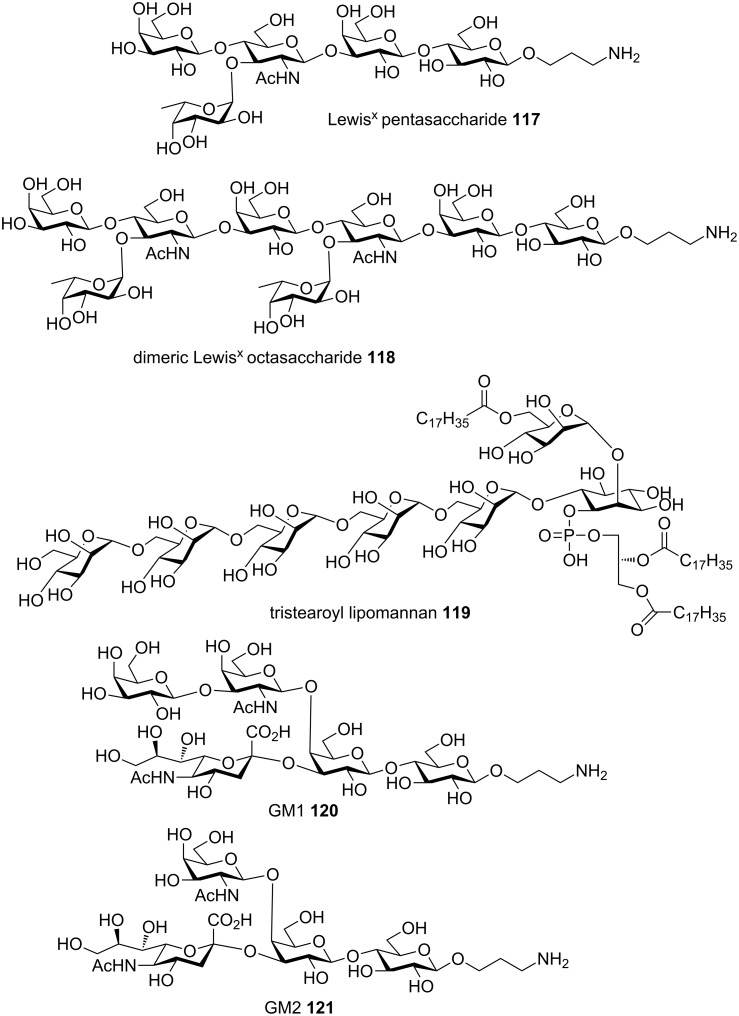
Representative complex glycans from glycolipid family synthesized by the preactivation-based thioglycoside method.

**Figure 6:**
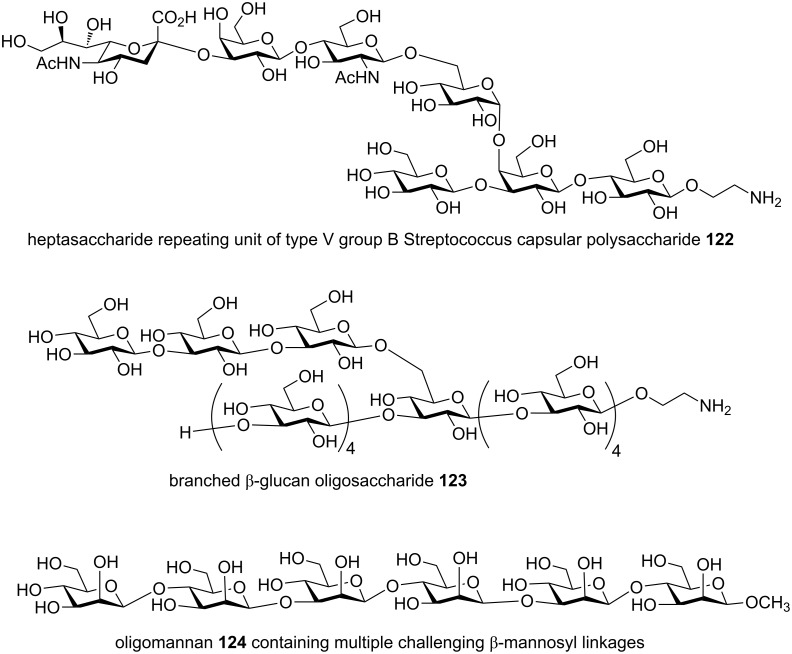
Representative microbial and mammalian oligosaccharides synthesized by the preactivation-based thioglycoside method.

**Figure 7:**
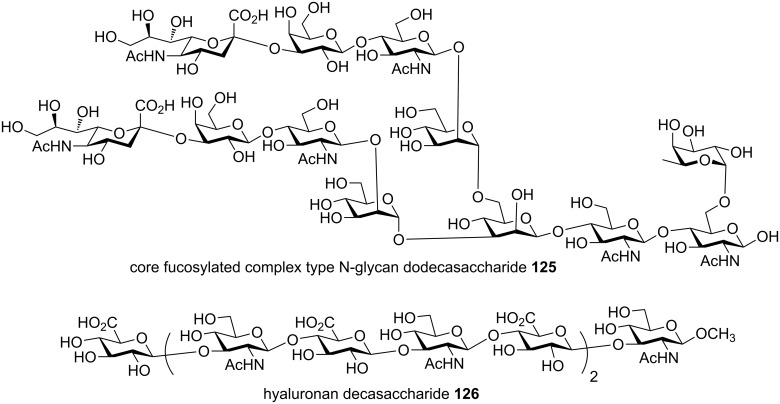
Some representative mammalian oligosaccharides synthesized by the preactivation-based thioglycoside method.

In addition to Globo-H **105** and arabinogalactan **116**, other complex oligosaccharides obtained by the preactivation-based thioglycoside method include branched oligosaccharides from glycolipid family including Lewis^X^ pentasaccharide **117**, dimeric Lewis^X^ **118** [63], tristearoyl lipomannan **119** [64], gangliosides GM1 **120** [65] and GM2 **121** (Figure 5) [66], microbial glycans such as the heptasaccharide repeating unit of type V group B Streptococcus capsular polysaccharide **122** [67], β-glucan oligosaccharides **123** from fungal cells [68–69], oligomannan containing multiple challenging β-mannosyl linkages **124** [54] (Figure 6), chitotetraose [70], mammalian glycans including complex type bisected N-glycan dodecasaccharide **125** [71], glycosaminoglycans including hyaluronic acid oligosaccharides **126** [72–73] (Figure 7), and heparan sulfate oligosaccharides including those bearing sulfate esters [74–75] and other sialylated glycans [76–77].

**Figure 8:**
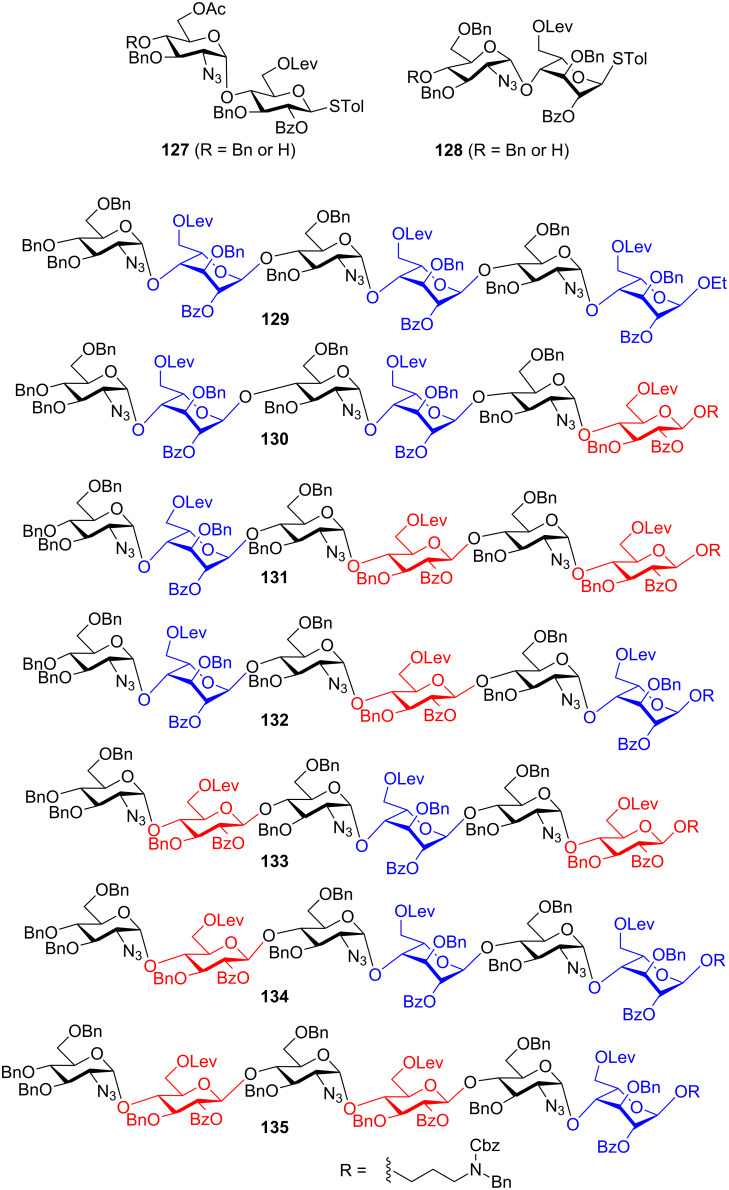
Preparation of a heparan sulfate oligosaccharides library.

As the preactivation-based glycosylation does not require the donor to have higher anomeric reactivity than the acceptor, this approach is particularly suitable for the synthesis of libraries of oligosaccharides by divergently combining building blocks. An example of this is the preparation of a library of heparan sulfate oligosaccharides (Figure 8) [74]. Alternating use of disaccharide building blocks **127** and **128** in preactivation-based one-pot glycosylation led to a panel of 7 heparan sulfate hexasaccharides **129**–**135** following the standard glycosylation protocol. The yields for one-pot glycosylation of all these hexasaccharides range from 50% to 70% highlighting the robustness of the protocol.

**Scheme 20:**
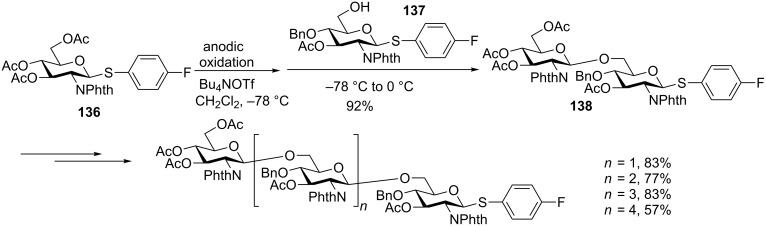
Synthesis of oligo-glucosamines through electrochemical promoted preactivation-based thioglycoside glycosylation.

Besides the more “classical” chemical activation of thioglycosides, Nokami, Yoshida and co-workers developed an alternative method taking advantage of electrochemistry for donor activation [59]. They have demonstrated that thioglycosides can be electrochemically oxidized in the presence of tetrabutylammonium triflate to yield a glycosyl triflate, which can be subsequently glycosylated. This approach has been adapted to an automated solution-phase synthesis of poly-β-D-(1-6)-*N*-acetylglucosamine [78]. The aryl group in arylthioglycosides was first optimized for both the donor and the acceptor, where the electron-withdrawing fluorine on the phenyl ring gave the best result. The thioglycoside donor **136** was preactivated through anodic oxidation, followed by the addition of the acceptor **137** to afford disaccharide **138** (Scheme 20). Repeating this process, a series of oligo-glucosamine ranging from tri- to hexa-saccharides **139**–**142** was successfully prepared.

**Scheme 21:**
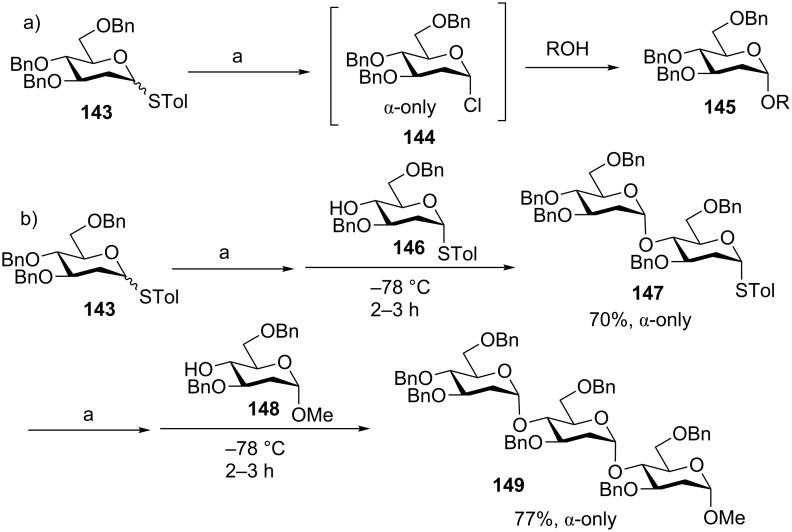
Synthesis of 2-deoxyglucosides through preactivation. Reagents and conditions: a) AgOTf, *p*-TolSCl, −78 °C.

2-Deoxy and 2,6-dideoxyglycosides are present in many natural products. Based on the preactivation protocol, the Wang group reported a stereoselective glycosyl chloride-mediated synthesis of 2-deoxyglucosides [79]. They found that the addition of AgOTf and *p*-TolSCl to donor **143** afforded the stable glycosyl chloride **144** as detected by NMR (Scheme 21a). The formation of the glycosyl chloride was possibly due to the presence of Lewis basic molecule sieves (MS 4 Å) in the reaction system lowering the reactivity of AgOTf [18]. As a result, *p*-TolSCl could directly activate the glycosyl donor forming glycosyl chloride due to the higher anomeric reactivities of deoxy glycosides compared to the corresponding pyranosides. Upon the addition of the acceptor, the glycosyl chloride could be activated by AgOTf producing the glycosylation product with good α selectivity. To test the applicability to iterative synthesis, donor **143** was preactivated with *p*-TolSCl and AgOTf at −78 °C followed by the addition of acceptor **146** to afford disaccharide **147** in 70% yield with complete α selectivity (Scheme 21b). This high α selectivity remained when disaccharide **147** was reacted with acceptor **148** to give trisaccharide **149** using the same promoter system.

**Scheme 22:**
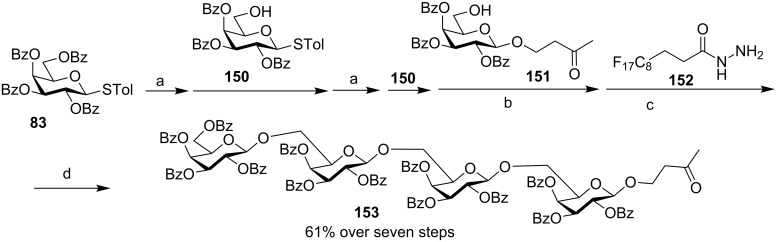
Synthesis of tetrasaccharide **153**. Reagents and conditions: (a) AgOTf, *p*-TolSCl, CH_2_Cl_2_, −78 °C; then **150**; (b) AgOTf, *p*-TolSCl, CH_2_Cl_2_, **150**, −78 °C to rt; (c) CH_2_Cl_2_/MeOH, then F-SPE; (d) acetone/trifluoroacetic acid, then F-SPE.

The preactivation-based one-pot approach can greatly accelerate oligosaccharide assembly. To facilitate isolation of the desired product from the reaction mixture, the Huang group reported a fluorine-assisted one-pot method, where no silica gel column chromatography was required [80]. To demonstrate the applicability of this method, a linear tetrasaccharide was synthesized bearing a ketone tag at the reducing end using building blocks **83**, **150** and **151** following the preactivation-based one-pot protocol (Scheme 22). After completion of the synthesis, a fluorinated hydrazide **152** was added to the reaction mixture to selectively “catch” the desired tetrasaccharide **153**, which was rapidly separated from non-fluorinated impurities by fluorous solid-phase extraction (F-SPE). Subsequent release of the compound from the fluorous tag and F-SPE yielded pure **153** in 61% overall yield from donor **83**.

**Scheme 23:**
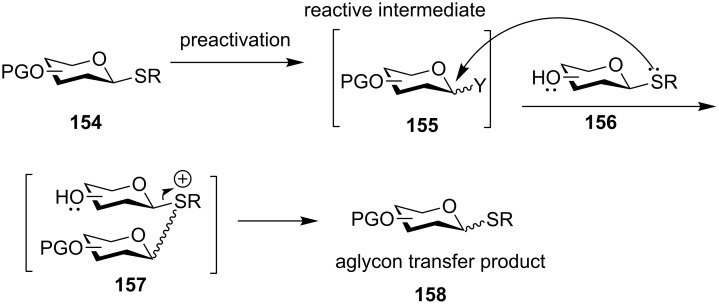
Aglycon transfer from a thioglycosyl acceptor to an activated donor can occur during preactivation-based glycosylation reaction. This side reaction can be suppressed by tuning the reactivity of acceptor aglycon or manipulating the reaction temperature.

One potential side reaction in using a thioglycosyl acceptor is the transfer of the thioaglycon of the acceptor to the activated donor presumably due to the high nucleophilicity of the aglycon compared with the hydroxy group of the acceptor (Scheme 23). Occasionally, the donor could be found regenerated upon addition of the acceptor following preactivation. This aglycon transfer phenomenon is not restricted to preactivation or thioglycosyl donors, as aglycon transfer products have been reported in premixed glycosylations with either glycosyl bromide or glycosyl trichloroacetimidate (Scheme 11b) [81–86]. The amounts of aglycon transfer products can be reduced by decreasing the nucleophilicity of the acceptor aglycon through steric effects [87] or tuning protective groups of acceptors [84,86], in some cases by lowering the reaction temperature [85].

## Conclusion

While conceptually simple, the temporal separation of donor activation and acceptor glycosylation in the preactivation protocol can enable chemoselective activation of the glycosyl donor without undesired acceptor activation. As a result, even an acceptor having higher anomeric reactivities than the glycosyl donor can be successfully glycosylated [18]. This protocol is found to be applicable to a wide range of glycosyl-donor types including thioglycosides, glycosyl sulfoxides, glycosyl hemiacetals, selenoglycosides, and 2-pyridyl glycosides. The newly formed oligosaccharide intermediate could be directly subjected to another round of preactivation and acceptor glycosylation without the need for additional synthetic operations to modify either protective groups or aglycon leaving groups. This can enable rapid glycan chain extension and improve overall synthetic efficiencies for glycan assembly.

Compared to the more traditional premixed method where both the glycosyl donor and the acceptor are present when the promoter is added, preactivation can generate reactive intermediates as the resting state allowing spectroscopic analysis such as low temperature NMR studies to better characterize the intermediate. This can help gaining a deeper insight into the reaction mechanism, which is critical for efforts to enhance the glycosylation yield.

The preactivation strategy is a powerful method for glycoassembly, which is evident from the successful synthesis of many complex oligosaccharides and glycoconjugates. However, glycosylation reactions are intrinsically sensitive to factors including protective groups on the glycan ring, reaction solvent, and additives present. As a result, further experimentation and analysis are needed to enable robust syntheses and achieve automation with comparable efficiencies of automated peptide and nucleic acid synthesis. With continuous development, the preactivation strategy will achieve wider applications in complex carbohydrate synthesis.
